# Determination of rotation center and diameter of femoral heads using off-the-shelf augmented reality hardware for navigation

**DOI:** 10.1038/s41598-024-64957-x

**Published:** 2024-07-04

**Authors:** Antoine Van Ravestyn, Taylor Frantz, Jef Vandemeulebroucke, Bart Jansen, Johnny Duerinck, Thierry Scheerlinck

**Affiliations:** 1 Department of Orthopedic Surgery and Traumatology, Universitair Ziekenhuis Brussel (UZ Brussel), Laarbeeklaan 101, 1090 Brussels, Belgium; 2 Vrije Universiteit Brussel (VUB), Research Group BEFY-ORTHO, Laarbeeklaan 103, 1090 Brussels, Belgium; 3 Department of Electronics and Informatics (ETRO), Vrije Universiteit Brussel (VUB), Pleinlaan 2, 1050 Brussels, Belgium; 4 IMEC, Kapeldreef 75, 3001, Leuven, Belgium; 5 Department of Neurosurgery, Universitair Ziekenhuis Brussel (UZ Brussel), Laarbeeklaan 101, 1090 Brussels, Belgium; 6 Department of Radiology, Universitair Ziekenhuis Brussel (UZ Brussel), Laarbeeklaan 101 1090 Brussels, Belgium

**Keywords:** Total hip arthroplasty, Computer aided navigation, Augmented reality, HoloLens, Surgery, Biomedical engineering, Bone, Cartilage, Skeleton, Osteoarthritis

## Abstract

In total hip arthroplasty (THA), determining the center of rotation (COR) and diameter of the hip joint (acetabulum and femoral head) is essential to restore patient biomechanics. This study investigates on-the-fly determination of hip COR and size, using off-the-shelf augmented reality (AR) hardware. An AR head-mounted device (HMD) was configured with inside-out infrared tracking enabling the determination of surface coordinates using a handheld stylus. Two investigators examined 10 prosthetic femoral heads and cups, and 10 human femurs. The HMD calculated the diameter and COR through sphere fitting. Results were compared to data obtained from either verified prosthetic geometry or post-hoc CT analysis. Repeated single-observer measurements showed a mean diameter error of 0.63 mm ± 0.48 mm for the prosthetic heads and 0.54 mm ± 0.39 mm for the cups. Inter-observer comparison yielded mean diameter errors of 0.28 mm ± 0.71 mm and 1.82 mm ± 1.42 mm for the heads and cups, respectively. Cadaver testing found a mean COR error of 3.09 mm ± 1.18 mm and a diameter error of 1.10 mm ± 0.90 mm. Intra- and inter-observer reliability averaged below 2 mm. AR-based surface mapping using HMD proved accurate and reliable in determining the diameter of THA components with promise in identifying COR and diameter of osteoarthritic femoral heads.

## Introduction

Total hip arthroplasty (THA) is one of the most frequent and successful procedures in orthopedics^1^. However, a good functional result depends on the correct restoration of normal joint kinematics^2^. As such, restitution of femoral version, offset, and leg length are key for a good clinical result^3–6^. Failing to reconstruct the native hip rotation center within 3–5 mm can have functional implications^7,8^. Yet, in clinical practice, global offset is not restored within 10 mm in 10% of patients, with some cases being over 25 mm^9,10^. Moreover, 16% to 32% of patients report leg length discrepancies over 10 mm^3^ following intervention.

Restoration of the original hip rotation center can be improved by pre-operative planning combined with infrared (IR) based surgical navigation^2,3^. But, even then, leg length discrepancies over 10 mm were seen in 10% of patients^3^. Moreover, most navigation systems are expensive, time-consuming, and cumbersome^11,12^. They also require tridimensional computer tomography (3D-CT) based preoperative templating, which adds to the cost and implies radiation exposure. Recent low-cost and portable augmented reality (AR) devices have been used as navigation systems for cup placement and aim to address these issues. However, such “consumer” AR systems have yielded mixed results and introduced errors due to attention shift and related to the fact that they were handheld devices, or had poor device localization performances^13–15^.

In non-deformed hips, failure to restore hip biomechanics is linked to surgical inaccuracy rather than to lack of implants^16^. We believe one source of surgical inaccuracy is the inability to locate the hip rotation center before resecting the femoral head and to keep it pinpointed once the femoral head has been removed. As such, we investigated the possibilities of a head-mounted augmented reality (AR) device, in combination with improved inside-out tracking, to estimate the femoral and acetabular hip rotation center during surgery, and to visualize it throughout the procedure.

## Materials and methods

The study comprised two parts, first a technical evaluation of the device’s ability to estimate the surface and diameter of a spheroid object using supplementary inside-out tracked stylus. For this, we used femoral head and cup prostheses as a baseline for performance. In the second part, we assessed the capacity of the device to perform the same estimation in addition to estimation of the femoral head rotation center using anatomical cadaver femurs.

### Head-mounted augmented reality device technology

The HoloLens 2 (Microsoft, Redmond WA, USA) is an off-the-shelf optical see-through head-mounted augmented reality device (AR-HMD) capable of rendering to the wearer computer graphics superposed with the real world. It can also sense the surroundings using a plurality of onboard sensors; red–green–blue (RGB), grayscale, infra-red (IR), and time-of-flight (ToF) depth camera. An AR application (app) was developed using the Unity game engine (Unity Technologies, San Francisco CA, USA) to project augmented reality overlays registered to real-world objects and to track passive reflective IR-labeled instruments using the onboard IR sensor^17,18^. As such, pointing an IR marked fine-tipped handheld stylus along the surface of objects allowed for the digitization of their surface coordinates within an IR-tracked reference frame. This reference frame provided a mechanism for the app to compensate for spatial drift of the AR-HMD—which can be up to several centimeters—as well as define a transformation between and the CT and AR devices, described later.

**Figure 1:**
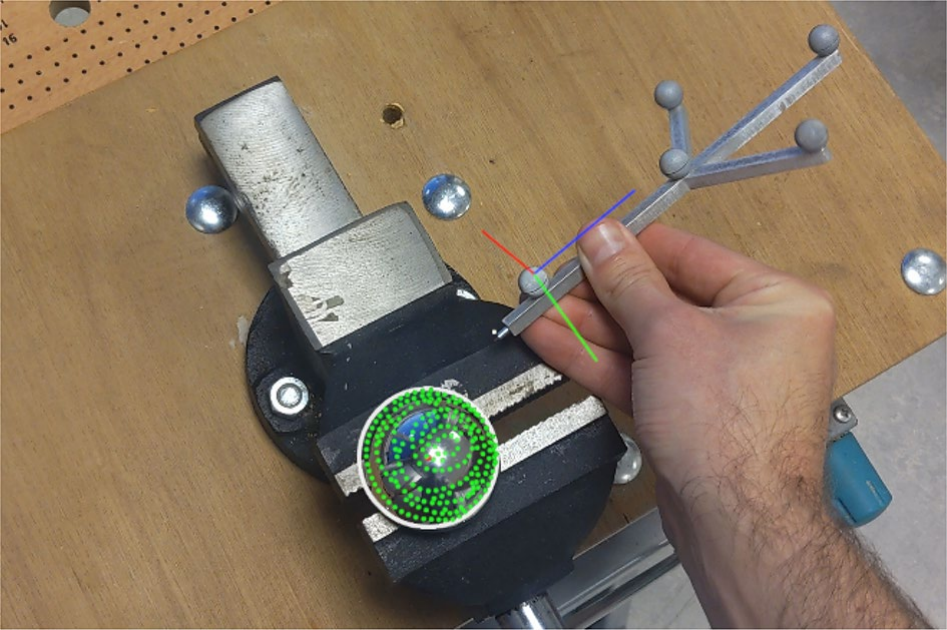
First person view of the AR app during system testing on prosthetics. The infrared tracked stylus is shown with its local coordinate system (red-blue-green axes). Sampled surface (green dots) and fit sphere (white silhouette) are shown overlaying the bench clamped prosthetic femur.

In the first part of the study, the AR device was used to estimate the center and surface diameter of 10 femoral prostheses and 10 acetabular cups of different diameters and materials (ceramic and metal) (Fig. 1). For this, the handheld IR-tracked stylus was used to sample points along the surface of each prosthesis, while performing a spiraling movement from the top of the sphere or the deepest point of the cup, towards the equator. In the second part of the study, the AR device was used to estimate the center of rotation and diameter of 10 femoral heads of cadaver specimens. Similarly, as before, we used the IR-tracked stylus to sample surface points in those areas that presented the least wear (medially and inferiorly) and avoided those areas with obvious wear (superiorly). As such, we tried to sample a sphere on the pristine aspect of the femoral head, without age-related wear. In both cases, after each measurement, a sphere was fit on the data using the fast geometric fit algorithm^19^, which has a reported improved speed and accuracy in fitting of noisy hemi- and semi-spherical point data when compared to classical methods. We calculated the distance from each of the surface coordinates to the fit sphere and used this to define outliers based on median absolute deviation^20^ and refit the data. The coordinates of the surface points were logged to the device for a posteriori analysis.

### Visualization through augmented reality

While pointing to the surface with the stylus, spheres corresponding to recorded coordinates were visualized through the HoloLens as a registered augmented reality overlay on the real world. After sphere fitting, we visualized the edge-outline and rotation center of the sphere to verify the fitment. A combination of the on-board HMD tracking (SLAM) and a fixed reference star equipped with IR markers allowed stabilization of the registered overlay, even when the headset moved compared to the object.

### Anatomical specimens and CT scanning

The study obtained ethical approval (BUN 1432021000443) from the Ethics Committee of UZ Brussel (Laarbeeklaan 101, 1090 Jette, Belgium), and informed consent was acquired from all study participants. The experiments were conducted in compliance with the International Council for Harmonisation of Technical Requirements for Pharmaceuticals for Human Use (ICH) Guideline for Good Clinical Practice (GCP). After ethical clearance, 10 dried human femurs were acquired from the anatomy department of the Vrije Universiteit Brussel.The femurs were chosen randomly, but specimens with excessive deformity were excluded. Each femur was fixed by radiolucent plastic straps (Kopp, Kahl am Main DE) to a wooden support board equipped with a fixed reference star of 4 infrared (IR) markers of 13 mm diameter (Brainlab, München DE).

The femurs anchored to the wooden board were CT scanned (Discovery CT750 HD, GE Medical systems) with the following settings: Helical mode, Peak voltage 140 kVp, tube current 80 mA, slice thickness 0.625 mm. From the CT data, 3D coordinates of the reference frame’s IR markers were obtained. This allowed a rigid transformation between the coordinate system of the reference frame in both the AR app and the CT image to be determined through least-squares fitting between corresponding marker coordinates in their respective coordinate systems^21^. In this way, data calculated from the AR app could be appropriately transformed into ground truth CT space.

### Finding the center of the femoral head in the CT scan dataset

**Figure 2:**
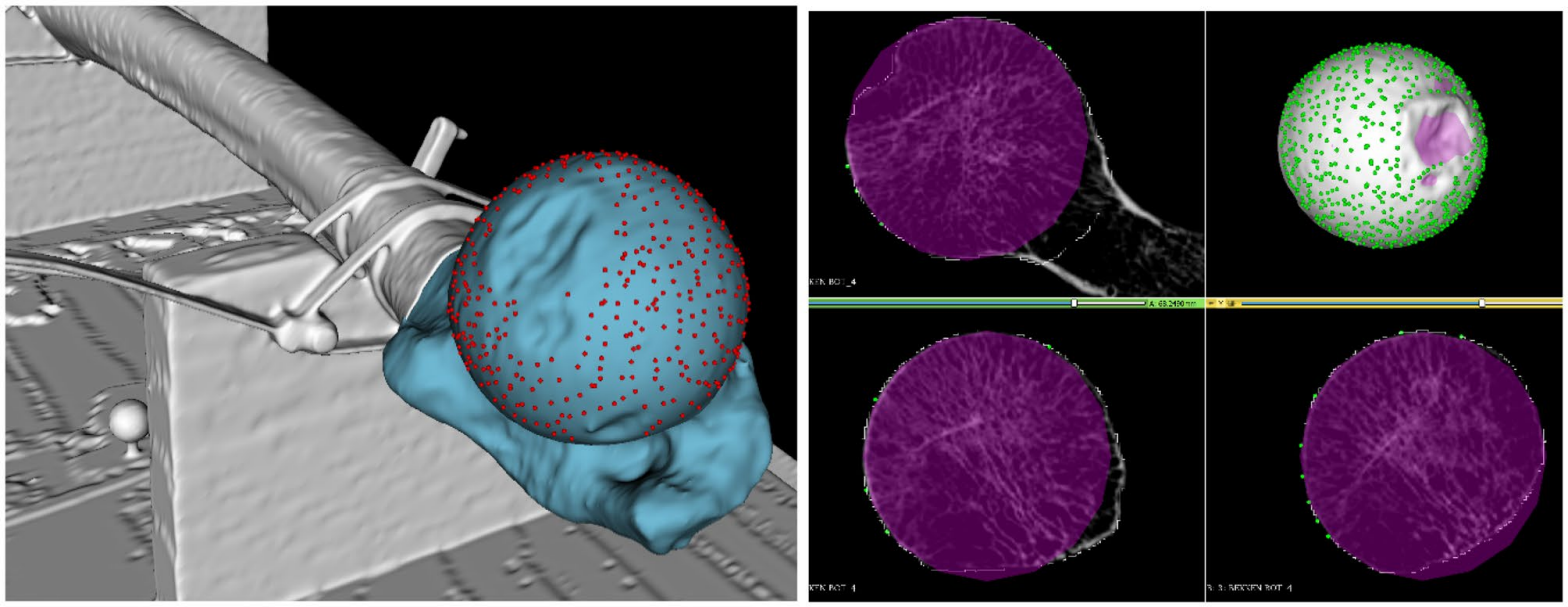
Left: Segmentation (blue) and surface sampling (red dots) of CT data used for ground truth definition. Right: Orthographic and 3D view of CT data (white) at the fit ground truth sphere (magenta) using the sampled points (green).

From the CT scan dataset, 3D slicer (http://www.slicer.org/ version 4.13.0)^22^ was used to segment the femoral bone using a combination of thresholding and manual correction. A point cloud along the segmented surface of the femoral head was sampled from the segmentation; taking care to avoid degenerative changes and the foveal margin. A sphere was then fit to the point cloud data, using the same technique as in this study’s first part, to estimate its center and diameter, these we considered as the ground truth [Fig. 2].

### Outcome measures and statistical analysis

During the first experiment, two observers (AVR, TF) measured twice, 10 spherical prosthetic heads and 10 cups with a Vernier caliper (Mitutoyo 530–122, Kawasaki, Japan) and with the head-mounted AR device. We report the accuracy of our AR measurements as the mean and standard deviation of the signed (relative) and unsigned (absolute) difference in diameter measured with both techniques.

To assess the intra- and inter-observer reproducibility of our AR-based tool, the diameter of spherical prosthetic heads and cups were similarly measured, by the same and by two different observers, respectively. We report the reproducibility and reliability results as the mean and standard deviation of the signed (relative) and unsigned (absolute) difference in diameter measurements.

During the second experiment, the same two observers assessed the femoral hip rotation center and the diameter of 10 femoral heads, 5 left and 5 right, using the AR device. These measurements were compared to the CT derived ground truth. We report positional errors of the rotation center as the signed distance between both sphere centers along an anatomical coordinate system defined by the CT scan as mediolateral (ML), anteroposterior (AP) and superoinferior (SI). Diameter errors are reported as the signed (relative) difference in diameters, where a negative value indicates the fit sphere was smaller than the ground truth. We report the results as means, standard deviations (SD), min–max, and 95% confidence intervals (CI_95_). As performed for the prosthetic heads and cups experiment, we investigated the reproducibility of our AR tool by comparing measurements between both observers.

All numerical analyses were carried out in Python (3.10; SciPy 1.11; NumPy 1.26).

## Results

### Accuracy and reproducibility measuring prosthetic heads and cups

The accuracy of the AR measurement workflow was assessed on 80 measurements performed by both observers on 10 prosthetic heads and 10 cups (Table 1). In this setting, the average error between the diameters measured with both methods was below 1.5 mm for both the head and cup. The maximal absolute error was less than 3 mm for the head and less than 4 mm for the cup. No statistical difference in absolute error was found between estimated cup and head sizes (t-test p = 0.07), however relative estimated diameter of prosthetic heads was found to be statically more undersized compared to the acetabular cups (t-test p = 0.03).

**Table 1:** Overall accuracy of the AR tool compared to measurements performed with a caliper by both observers.

*N* = *80*	Relative error [mm]		Absolute error [mm]	
	Head	Cup	Head	Cup
Mean	− 1.22	− 0.67	1.37	1.11
SD	0.96	1.33	0.73	0.98
Min	− 2.74	− 3.83	0.21	0.09
Max	0.72	3.86	2.74	3.86
CI_95_	[− 1.52– − 0.92]	[− 1.10– − 0.28]	[1.14–1.60]	[0.81–1.41]

Negative values indicate that the AR-based sphere was smaller than the ground truth. N is the total number of measurements used in the calculation.

AR-based measurement of the diameter of a perfect sphere and cup were performed twice by the same observer [Table 2] and by two different observers [Table 3]. The average intra- and interobserver absolute errors were below 2 mm in all cases with standard deviations below 1.5 mm. In both settings, mean absolute measurement errors between prosthetic heads and cups were not statistically different (t-test intra-observer, p = 0.10; t-test inter-observer, p = 0.36).

**Table 2:** Intra-observer reliability for repeated measurements performed by the same observer (AVR) using the AR based technique.

*N* = *40*	Relative error [mm]		Absolute error [mm]	
	Head	Cup	Head	Cup
Mean	0.08	− 0.10	0.81	0.70
SD	0.97	0.96	0.54	0.67
Min | Max	− 1.63 | 1.32	− 2.23 |1.54	0.00 | 1.63	0.01 | 2.23
CI_95_	[− 0.52–0.68]	[0.69–0.49]	[0.48–1.14]	[0.29–1.11]

N is the total number of measurements used in the calculation.

**Table 3:** Inter-observer reliability for measurements performed by two different observers (AVR and FT) using the AR based technique.

*N* = *40*	Relative error [mm]		Absolute error [mm]	
	Head	Cup	Head	Cup
Mean	− 1.08	− 0.47	1.57	1.13
SD	1.63	1.30	1.18	0.79
Min | Max	− 5.32 | 1.34	− 2.93 | 3.05	0.14 | 5.32	0.10 |3.05
CI_95_	[− 1.80*–*− 0.36]	[− 1.04–0.10]	[1.05*–*2.09]	[0.78–1.48]

N is the total number of measurements used in the calculation.

### Accuracy of measurements performed on anatomical femurs

**Figure 3:**
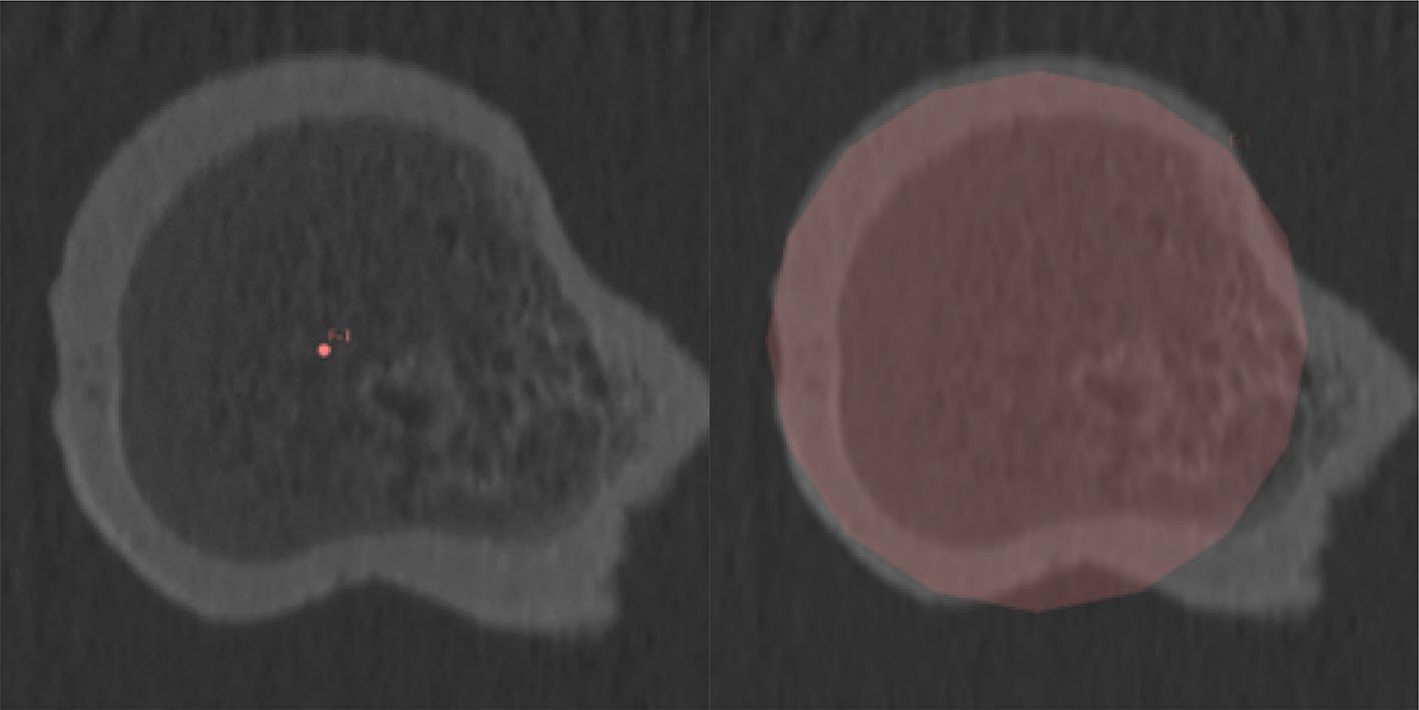
Estimated center of rotation (left) and diameter (right) of an anatomical femur using the AR device, mapped to the ground truth CT data for a single experiment.

**Figure 4:**
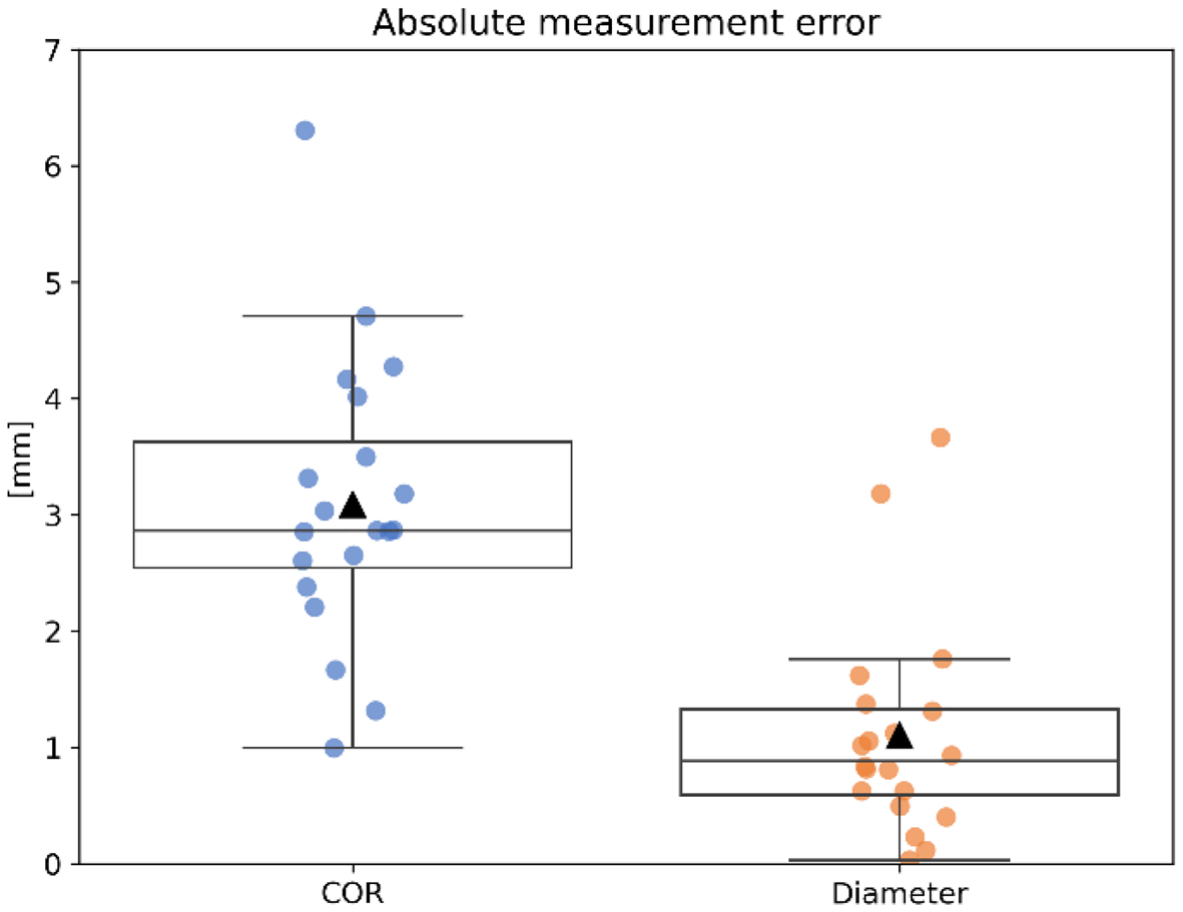
Absolute error between the estimated and ground truth position of the femoral COR and head diameter.

To evaluate the accuracy of the AR surface identification technique, we calculated the distance between the centers of rotation (COR) and the difference in diameters of 10 human femoral heads obtained through surface scanning and from the CT scans. Figure 3 illustrates the fit quality of a single measurement with respect to its ground truth CT scan. Absolute errors over all experimental data are illustrated in Fig. 4 and reported in Table 4.

**Table 4:** Error between estimated and ground truth femoral COR position and head diameter.

*N* = *20*	COR [mm]	Diameter (relative) [mm]	Diameter (absolute) [mm]
Mean	3.09	− 0.66	1.10
SD	1.18	1.26	0.90
Min | Max	0.99 | 6.30	− 3.66 | 1.37	0.03 | 3.66
CI_95_	[2.52–3.66]	[− 1.26–− 0.05]	[0.67–1.53]

N is the total number of measurements used in the calculation.

**Figure 5:**
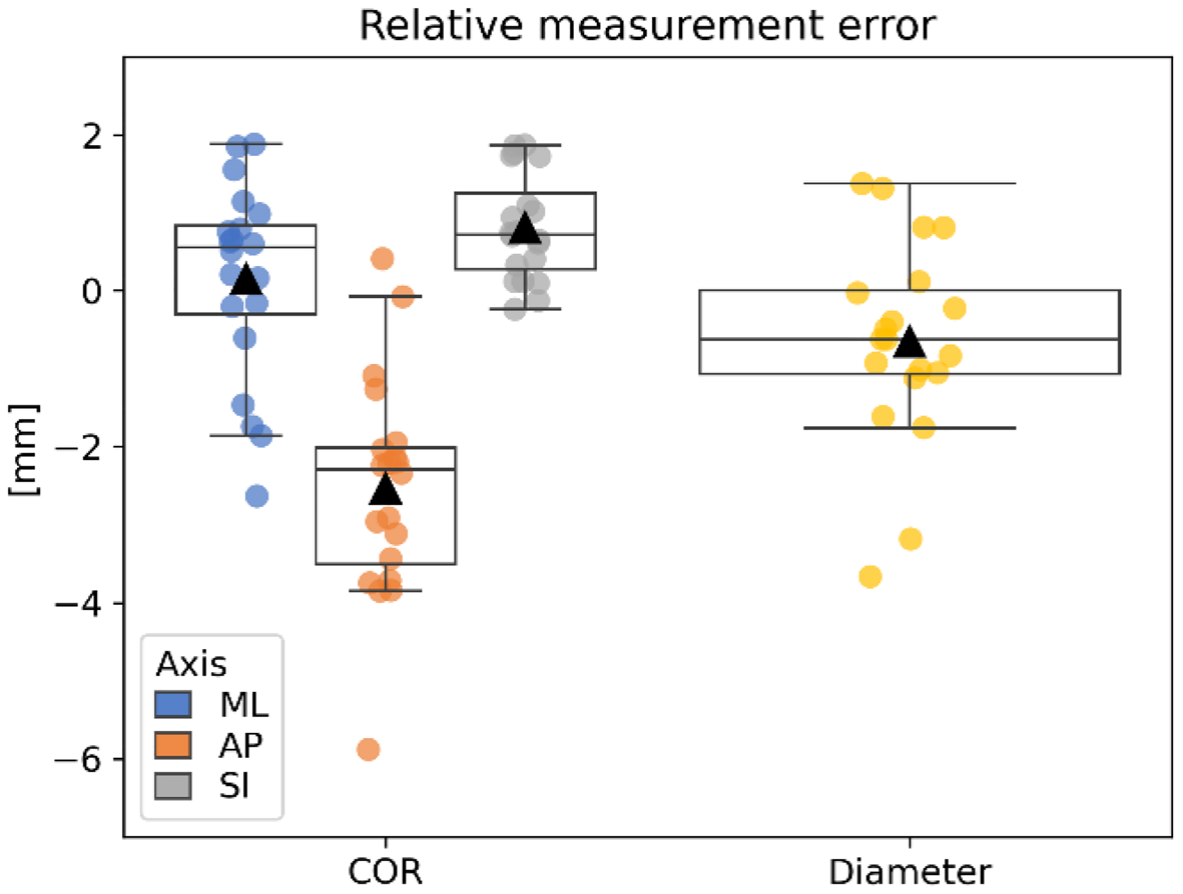
Difference in estimated center of rotation (COR) vector along three anatomical axes: ML (mediolateral), AP (anteroposterior) and SI (superoinferior) as well as signed differences in diameter.

The decomposition of the positional error of the COR along the mediolateral (ML), anteroposterior (AP) and superoinferior (SI) is illustrated in Fig. 5 along with the relative diameter error. An analysis of variance (ANOVA) revealed that differences between axes were significant (p < 0.01). A t-test, with Bonferroni correction (alpha = 0.017) for three measurements, showed that the anteroposterior error was statistically different from both the mediolateral axis (p < 0.01) and the superoinferior axis (p < 0.01) There was no statistical difference in errors along the mediolateral and superoinferior axes (p = 0.047).

### Reproducibility of measurements performed on anatomical femurs

The mean inter-observer variability of AR measurements of the femoral head diameter was below 2 mm. For the location of the COR this was fractionally over 2 mm (Table 5). There was no statistical difference between observers in either relative diameter (t-test p = 0.81), absolute diameter (t-test p = 0.80), or rotation center estimation (t-test p = 0.17). There was no statistical difference found in inter-observer variability (Table 6) based on diameter estimation (t-test p = 0.70).

**Table 5:** Inter-observer variability between observers (AVR and FT) for AR surface scanning of femoral heads.

*N* = *20*	COR [mm]	Diameter (relative) [mm]	Diameter (absolute) [mm]
Mean	2.08	0.15	1.55
Standard deviation	0.82	2.00	1.27
Min | Max	1.19 | 4.26	− 4.47 | 2.25	0.14 | 4.47
CI_95_	[1.47–2.70]	[− 1.36–1.66]	[0.59–2.51]

N is the total number of measurements used in the calculation.

**Table 6:** Intra-observer variability among observer (AVR) for AR surface scanning of femoral heads.

*N* = *20*	Diameter (relative) [mm]	Diameter (absolute) [mm]
Mean	0.92	1.20
Standard deviation	1.21	0.94
Min | Max	− 1.22 | 2.76	0.17 | 2.76
CI_95_	[0.01–1.84]	[0.50–1.91]

N is the total number of measurements used in the calculation.

## Discussion

Surface scanning using a head-mounted AR device with a tracked handheld stylus has shown promising results in determining the diameter of prosthetic femoral heads and acetabular cups. The intra- and inter-observer reliability of this technique was below 2 mm, indicating good accuracy and reliability. The measurements of acetabular cups had larger inter- and intra-observer errors. We attributed this to difficulties in maintaining the rounded tip of the stylus perpendicular to the narrow concave surface of the cup. As such, a sharper stylus tip could improve accuracy and should be considered in future applications.

Applied to cadaveric femoral heads, the stylus-based surface scanning technique demonstrated an acceptable accuracy in determining the center of rotation (mean error: 3.1 mm) and the head diameter (mean absolute error: 1.1 mm). Differences in locating the COR were attributed to our inability to select the same sampling region during surface scanning and within the CT scan datasets. On average, our AR-based method underestimated the femoral head diameter by 0.66 mm.

Finding the center of rotation in an accurate and reproducible way is crucial in hip arthroplasty, as failing to restore or correct that rotation center can have functional consequences. Our AR, stylus-based, surface scanning technique successfully achieved that objective, as the CI_95_ was below 5 mm; a value cited in literature as a threshold to prevent functional complications^7,8^.

Besides the ability to find the hip rotation center, the AR-HMD is able to show it as a registered overlay on the operating field even after head resection. As such, the surgeon could have continuous visual feedback during the different stages of hip arthroplasty surgery, i.e. femoral broaching, stem trial, final stem insertion and choosing the neck length. As such, the system could provide feedback independently of the stem that is used. Alternatively, it could be combined with a calibrated implant-specific broach and stem navigation system. This would allow proper AR-based hip stem navigation, as the relation between both, the original and the prosthetic rotation center, could be visualized. A similar approach, using a stylus and an AR-HMD device, could be used to scan the inner surface of the acetabulum and to find the acetabular rotation center. If proven accurate and valid, this technique, combined with a reference star fixed to the pelvis, could allow navigating both, acetabulum reaming and cup insertion. As such, the proposed technique would offer the possibility to navigate the full hip arthroplasty process.

Compared to other navigation systems^11,12^, we used a small, low-cost, off-the-shelf AR-HMD and, adapted it with an inside-out tracked IR marked reference star and stylus. This, combined with implant independency and improved intra-operative ergonomics^23^, could favor fast adoption in clinical practice. Furthermore, the ability to locate the center of rotation with a mean error of 3.1 mm makes it more accurate than one of the major robotic-assisted solutions, i.e. the MAKO robot, which achieves restoration of the hip joint with changes in COR up to 10 mm, in most cases^24^. Other studies examining the accuracy of hip joint restoration mostly look at the center of rotation on the acetabular side^25^ or focus on leg length discrepancies and femoral offset. Importantly, when compared to traditional templating, the proposed AR workflow does not require preoperative imaging. Moreover, it can be used “on-the-fly” intraoperatively, reducing the preoperative workflow. Although unmeasured during experimentation, the time required for a single surface templating, being not more than about a minute, is not seen as negative to interventional workflow.

On the other hand, the AR-HMD’s display focal distance of two meters, might lead to perceptible visual difficulties like focal rivalry and vergence-accommodation conflict^26,27^. Additionally, the high levels of auditory noise typically present during orthopedic surgical procedures could affect the voice commands used during AR-based navigation^13^. These drawbacks should be investigated in a cadaver setting simulating a real intraoperative situation, before considering its use in clinical practice. In a practical use case, sometimes, the lack of occlusion-based depth cues from the AR device’s display technology results in the AR content not being visually integrated into the environment in a natural way. It was found during development, that presenting minimal visual data improved, though did not eliminate, this. For example, rather than displaying the templated femoral head as a sphere, a simple 2D contour around its equator with a small centered red dot for the rotation center was ideal (Fig. 1).

Our method is most suitable when the femoral or prosthetic head is dislocated when still fixed to the femur as through a posterior approach or in revision cases. Our technique is difficult to apply when the femoral neck is cut before hip dislocation, as during an anterior hip approach. In that case, the original hip rotation center could be estimated through the small head surface visible along the acetabular rim or using a functional dynamic method based on pivoting the hip within the acetabular cavity.

Post hoc analysis of sphere fit data as calculated using the fast geometric fit algorithm^19^ when compared to prominent least-squares techniques^28^ showed less than 0.05 mm difference in center or diameter. However, as care was made to sample a non-degenerate femoral surface, this reduced noise. In the presence of greater measurement noise, or a more limited sample area, as encountered during THA anterior approach, the adopted method may be more advantageous.

The limitations of our study include the small sample size, the selection of femoral heads that were not severely deformed and the experimental setup that did not simulate a surgical setting. As such, and before adoption in clinical practice, we recommend evaluating our technique in cadaver experiments simulating real-life surgery. In such a study, the outcome measures should not only include the accuracy to determine the hip rotation center, but also the ability of our technique to guide surgeons. Therefore, such a study should evaluate the capacity of AR guidance to position hip implants according to a predefined target and compare it to freehand techniques and/or current navigation systems.

## Conclusion

In conclusion, our stylus-based surface scanning technique using an off-the-self head-mounted AR device is an accurate and reliable method to find the diameter of prosthetic femoral heads and acetabular cups. It also showed promising results in finding the center of rotation and the diameter of human femoral heads in an experimental setting. With its low cost, compactness and ease of use, this technique could improve the accuracy of hip arthroplasty procedures. However, further research and validation are needed to explore its applicability in a clinical setting.

## Data Availability

The datasets generated during and/or analyzed during the current study are available from the corresponding author upon reasonable request.
